# Health Diplomacy in Limited‐Resource Settings in Africa: What It Takes Towards a Realistic, Resilient and Equitable Agenda?

**DOI:** 10.1002/puh2.70381

**Published:** 2026-10-22

**Authors:** Majani Edward, Ritha Anderson Willilo, Godfrey Sama, Ntuli A. Kapologwe

**Affiliations:** ^1^ Research and Training Unit, Department of Research and Innovation MacWish College of Health and Allied Sciences Mwanza Tanzania; ^2^ Centre for Reforms, Innovation, Health Policies and Implementation Research Dodoma Tanzania; ^3^ Bergen Centre of Ethics and Priority Setting University of Bergen Bergen Norway; ^4^ East, Central and Southern Africa Health Community Arusha Tanzania

**Keywords:** equitable agenda, health diplomacy (HD), health systems strengthening, local ownership, resource‐limited settings

## Abstract

Health diplomacy (HD) in African nations is often characterized as limited settings due to resource scarcity, weak governance and fragmented health systems. Although HD is crucial for addressing the continent's interconnected health challenges, including endemic diseases, infectious threats, antimicrobial resistance (AMR), climate change, persistent conflicts and foundational weaknesses, its effectiveness is frequently undermined by the structural persistence of hidden power, a legacy of colonialism that controls agenda‐setting and perpetuates dependency. Drawing on decolonial theory, this article argues that a purely technical application of HD is insufficient. It proposes a strategic framework for HD implementation that builds long‐term resilience and sustainability. Achieving a realistic agenda requires a commitment to local ownership and co‐leadership, health systems strengthening (HSS) and context‐specific adaptation. Simultaneously, an equitable agenda must proactively tackle power imbalances, prioritize a rights‐based approach and enhance local agency in diplomatic negotiations. By strategically linking health initiatives to broader security/development goals (smart power) and framing health as a fundamental human right, this framework enables HD to transition from a mechanism that risks reinforcing inequalities to a powerful tool for achieving resilient, sustainable, and truly equitable collective health security.

## 1 Introduction

Health challenges in resource‐limited settings increasingly transcend national borders and demand coordinated diplomatic responses that extend beyond conventional public health interventions [1]. Across many African countries, the health landscape is shaped by a complex interaction of endemic diseases, emerging and re‐emerging infectious threats [2], antimicrobial resistance (AMR), climate‐related health disruptions, conflict, migration and longstanding health system weaknesses. These interconnected realities reinforce the understanding that health outcomes are no longer determined exclusively within national boundaries but are increasingly influenced by regional and global governance structures.

Health diplomacy (HD) has consequently emerged as an important mechanism through which governments, regional institutions, international organizations and non‐state actors negotiate collective responses to shared health challenges [3]. Broadly, HD refers to the processes through which health concerns are incorporated into foreign policy, international negotiations and multilateral cooperation to improve population health outcomes while advancing broader political and development goals. In the African context, HD represents an increasingly important strategy for harmonizing policies, mobilizing resources, strengthening institutions and fostering regional health security [4].

However, despite increasing investment in HD, implementation within limited‐resource settings continues to face persistent structural barriers [5]. These settings are frequently characterized by constrained domestic financing, dependence on external aid, fragmented governance systems, workforce shortages and weak institutional capacity. Such conditions frequently expose nations to unfair bargaining contexts where national ambitions are shaped by external priorities, and long‐term viability is jeopardized.

Understanding these limitations requires moving beyond purely technical explanations. Decolonial scholarship argues that historical and contemporary forms of power continue to influence global health governance through mechanisms that extend beyond formal political structures [6]. Hidden and invisible forms of power may determine who participates in negotiations, which priorities receive attention, and whose interests ultimately shape outcomes [7]. Therefore, HD may unintentionally reproduce dependency rather than promote genuine partnership and local agency.

Recent experiences further illustrate these tensions. During the COVID‐19 pandemic, global and regional responses revealed persistent inequalities in access to medicines, mobility restrictions, vaccine allocation and decision‐making authority [8]. Emerging evidence also suggests that diplomatic responses often insufficiently incorporate patient perspectives and rights‐based considerations during crises. Qualitative evidence among health diplomats has demonstrated gaps in understanding of patient rights frameworks and limited institutional preparation for integrating community‐level health priorities into diplomatic processes [9]. These observations suggest the need to strengthen patient‐centred and rights‐based dimensions within contemporary HD.

At the same time, Africa continues to experience growing opportunities for regional collaboration through institutions, such as the African Union and regional health communities [10]. Ongoing diplomatic priorities, including AMR governance, workforce mobility, surveillance coordination, emergency preparedness and regulatory harmonization, demonstrate increasing recognition that resilient health systems require collective action rather than isolated national responses.

Against this background, this article develops a conceptual framework for HD implementation in resource‐limited settings. Drawing primarily on decolonial theory and complementary perspectives from global governance and institutional cooperation, the article proposes pathways for advancing realistic, resilient and equitable health agendas through local ownership, health systems strengthening (HSS), patient‐centred approaches and strengthened regional agency. Although conceptual in nature, this framework seeks to provide policy‐relevant guidance while identifying areas requiring future empirical evaluation.

## 2 Methodology

This article adopts a conceptual analysis approach to develop a framework for implementing HD in resource‐limited settings. Rather than generating primary empirical data, the article synthesizes contemporary literature from global HD, decolonial scholarship, HSS and international relations to examine how power structures influence diplomatic practice and health governance.

The framework was developed through an interpretive review of peer‐reviewed literature and major policy documents relating to global health governance, decolonial theory, HSS and African regional health cooperation. Particular attention was given to studies examining power asymmetries, diplomatic negotiations, local ownership, health security and regional institutional collaboration. These bodies of literature were analysed comparatively to identify recurring concepts, implementation challenges and strategic approaches that could be integrated into a coherent conceptual framework.

The literature search was conducted across PubMed/MEDLINE, Scopus, Web of Science and Google Scholar, supplemented by manual searches of policy repositories, including the World Health Organization, the African Union and Africa Centres for Disease Control and Prevention. Search terms combined, using Boolean operators, the concepts of ‘HD’, ‘global health governance’, ‘decolonial theory’, ‘hidden power’, ‘HSS’, ‘local ownership’, ‘patient‐centred’, ‘rights‐based approach’ and ‘Africa’, together with related terms, such as ‘resource‐limited settings’ and ‘regional health cooperation’. Searches covered literature published between 2015 and 2026, reflecting the period of accelerated scholarly and policy attention to decolonial critiques of global health and to HD following major regional and global health security reforms. Records were included if they were peer‐reviewed journal articles, book chapters or major policy documents published in English that substantively addressed HD, decolonial theory, HSS or African regional health governance; items were excluded if they addressed diplomacy in non‐health domains, were non‐peer‐reviewed commentary without conceptual contribution or were inaccessible in full text. Screening and thematic synthesis were conducted iteratively by the author team, with recurring concepts, tensions and strategic approaches extracted and organized into the domains presented below.

Consistent with established approaches to conceptual research, the objective of this article is not to evaluate intervention effectiveness but to organize existing theoretical knowledge into a practical framework capable of informing policy development, implementation and future empirical research. Illustrative examples from African regional health initiatives and global HD are incorporated to demonstrate the potential application of the proposed framework.

As a conceptual study, the framework should be viewed as a theory‐informed model requiring future empirical validation through comparative case studies, implementation research and policy evaluation across diverse resource‐limited settings.

## 3 Conceptual Framework

Understanding how HD can generate realistic, resilient and equitable outcomes in resource‐limited settings requires an analytical framework that captures both institutional processes and underlying power relations (Table 1, Figure 1) [11]. Although international relations theories provide useful explanations for cooperation and negotiation, this article primarily draws on decolonial theory and complementary institutional perspectives to explain why HD outcomes frequently remain uneven across low‐resource contexts.

**TABLE 1:** Key concepts table.

Concept	Definition	Operational manifestation
Hidden power	Control of agenda‐setting	Donor conditionalities
Local ownership	National leadership in policy	Domestic financing, co‐design
Smart power	Linking health to foreign policy	Pandemic preparedness negotiations
Equitable agenda	Fair participation and benefit	Inclusive governance

*Source:* Prepared by the authors for this publication.

**FIGURE 1:**
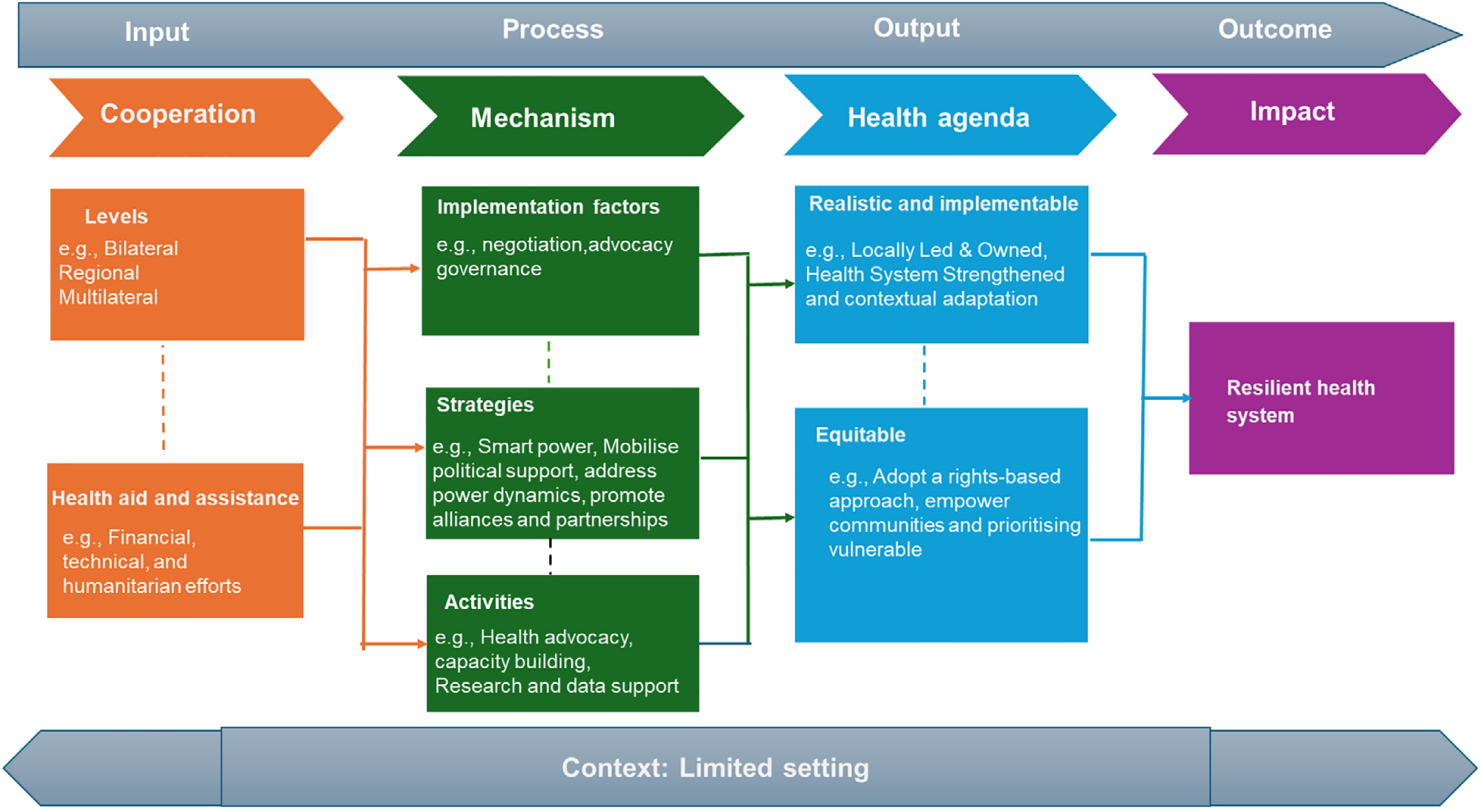
Conceptual framework in limited‐resource settings. *Source:* Prepared by the authors for this publication.

This choice of lens is not uncontested. Realist accounts of international relations would explain uneven HD outcomes primarily as a function of unequal material capabilities and national interest maximization, rather than of colonially rooted discursive and institutional power; from this perspective, resource‐limited states are disadvantaged bargainers not because of inherited structures of domination but because of ordinary asymmetries in economic and military power. Liberal institutionalist perspectives, by contrast, emphasize that multilateral institutions, regimes and repeated cooperation can progressively narrow these asymmetries through rules, transparency and reciprocity, a more optimistic reading of institutional reform than decolonial theory typically allows. Some scholars integrating global health security and universal health coverage through governance reform, likewise foreground institutional design and coordination failures over historically, inherited power relations [16]. This article retains decolonial theory as its primary lens because it best accounts for the persistence of unequal agenda‐setting even where formal institutional rules are ostensibly neutral, but it treats realist and liberal institutionalist insights as complementary rather than incompatible: Material capability gaps and weak institutional design are treated here as mechanisms through which hidden and invisible power operate, rather than as alternative, mutually exclusive explanations.

Decolonial theory provides a critical lens for understanding how historical and contemporary structures continue to shape global health governance [12]. Colonialism has extended beyond territorial domination into enduring systems of political, financial, epistemic and institutional influence that continue to affect decision‐making authority and resource allocation [12]. Within contemporary HD, these legacies often operate through unequal negotiating positions and externally driven policy priorities.

To operationalize this perspective, the framework adopts the typology of visible, hidden and invisible power. Visible power refers to formal decision‐making structures, governance mechanisms and publicly observable negotiations. Hidden power operates through agenda‐setting processes that determine who participates in negotiations and which issues receive institutional attention [13]. Invisible power shapes beliefs, norms and assumptions that influence how actors perceive legitimacy, authority and acceptable policy options [14].

In resource‐limited settings, hidden and invisible forms of power may contribute to sustained dependence by privileging donor priorities, externally defined indicators and short‐term programmatic objectives over nationally determined health goals [15]. These mechanisms may unintentionally weaken local ownership and reduce opportunities for institutional development.

At the same time, diplomacy should not be viewed exclusively as an instrument of power asymmetry. HD can also function as a corrective mechanism that enables countries to negotiate more equitable arrangements, strengthen domestic systems and promote collective action [16]. Diplomatic engagement creates opportunities to reshape agenda‐setting processes through co‐leadership models, regional institutions and evidence‐informed negotiations.

Recent experiences demonstrate the practical implications of these dynamics. During the COVID‐19 response, competing diplomatic approaches revealed tensions between solidarity‐based health cooperation and geopolitical interests [17]. Similar patterns continue to emerge in negotiations related to AMR, access to medical technologies, workforce migration and emergency preparedness [18]. These examples suggest that diplomatic success cannot be measured solely by agreements reached but also by whether outcomes strengthen local systems and improve population access.

Importantly, emerging evidence indicates that HD frameworks have not consistently incorporated patient perspectives. Studies examining HD practice have reported limited awareness of patient rights frameworks among diplomatic actors and inadequate mechanisms for incorporating community‐level needs during crises [19]. Such findings highlight the need to move beyond state‐centred diplomacy towards patient‐centred and rights‐based approaches that ensure equitable access and continuity of care.

Accordingly, the conceptual framework proposes that HD in limited‐resource settings should operate across four interdependent domains.

These four domains were derived inductively from the comparative literature synthesis as described in the methodology: Local ownership and co‐leadership, and HSS, recur consistently as the structural conditions under which diplomatic agreements translate into durable institutional capacity rather than short‐lived programmatic gains; context‐sensitivity recurs as the mechanism explaining why identical diplomatic templates succeed in some settings and fail in others; and patient‐centred, rights‐based principles recur as the corrective most directly responsive to the hidden‐power dynamics identified through the decolonial lens. The four domains are therefore treated as interdependent rather than additive: Weakness in one (e.g., externally driven agenda‐setting) tends to undermine progress in the others, which is why the framework presents them as a single interlocking agenda rather than a checklist of independent reforms.

First, diplomatic engagement should support local ownership and co‐leadership by positioning national institutions and regional actors as agenda‐setters rather than recipients of externally designed interventions.

Second, diplomacy should prioritize HSS, emphasizing investments that improve governance, workforce development, financing mechanisms, service delivery capacity and long‐term institutional resilience.

Third, implementation must remain context‐sensitive, recognizing differences in political environments, epidemiological profiles and social realities.

Fourth, diplomacy should embed patient‐centred and rights‐based principles that promote accountability, community participation, equitable access and protection of vulnerable populations.

Collectively, these dimensions provide the foundation for a realistic, resilient and equitable HD agenda capable of supporting sustainable health outcomes in resource‐limited settings.

## 4 What It Takes Towards a Realistic, Resilient and Equitable Agenda

Translating HD into meaningful outcomes in resource‐limited settings requires moving beyond technical cooperation towards a deliberate transformation of governance structures, negotiating practices and implementation approaches. A realistic and equitable agenda depends not only on diplomatic engagement itself but also on how diplomacy redistributes influence, strengthens institutions and responds to community priorities.

A central requirement is strengthening local ownership and co‐leadership. Effective HD should enable national governments, regional institutions and local technical actors to define priorities, shape implementation pathways and monitor outcomes. Co‐leadership reduces dependency and creates conditions for sustained institutional development. This shift requires diplomatic arrangements that prioritize shared accountability rather than donor‐driven implementation.

Equally important is a sustained commitment to HSS. Diplomatic success should not be evaluated exclusively by immediate outputs, such as funding commitments or emergency responses, but by whether agreements generate durable improvements in governance, financing, infrastructure, workforce capacity, supply chains and service delivery. Regional examples demonstrate that coordinated action can support these goals through workforce mobility agreements, harmonized regulation and collaborative learning platforms.

The third requirement is context adaptation. HD strategies cannot be transferred uniformly across settings. Successful implementation depends on alignment with local governance arrangements, disease burdens, political realities and institutional capacities. Flexible approaches improve feasibility and reduce implementation failure associated with externally imposed models.

Another important consideration is acceptance of gradual progress. Structural inequities in global health governance cannot be transformed through short‐term interventions. Incremental gains—including improved negotiation capacity, stronger regional institutions and increased domestic leadership—may represent meaningful markers of sustainable progress.

Achieving resilience also requires confronting inherited and contemporary power asymmetries. HD should actively identify mechanisms through which hidden power shapes agenda‐setting, financing decisions and representation. Greater transparency, inclusive negotiation structures and strengthened domestic capacity can improve equity in diplomatic processes [20].

A revised rights‐based approach further strengthens this agenda. Health should be treated not solely as a matter of security or economic productivity but as a fundamental human right. Diplomatic initiatives should therefore protect continuity of care, preserve access to essential services during emergencies and integrate accountability mechanisms that monitor whether agreements benefit affected populations.

The operational costs of neglecting these rights‐based and communicative dimensions were made explicit during the COVID‐19 pandemic. Analyses of the global response have argued that inconsistent international messaging; fragmented decision‐making among global, regional and national actors and inadequate coordination of public communication undermined public trust and produced policy incoherence, exposing the political, ethical, legal and medical fault lines of contemporary HD [21]. This experience illustrates the operational dimension of resilience proposed here: A rights‐based agenda is incomplete unless it is matched by deliberate investment in coordinated, transparent risk communication across diplomatic and technical levels, as communication failure can erode public trust as quickly as resource scarcity or governance weakness.

To operationalize this principle, this article advances a patient‐centred HD perspective. Patient‐centred diplomacy recognizes individuals and communities as legitimate stakeholders in global and regional health governance [22]. This approach promotes community engagement, responsiveness to lived experience and protection of healthcare access during crises. Incorporating patient voices provides an important safeguard against policies that privilege geopolitical objectives over public need.

Finally, future‐oriented HD should respond to emerging priorities shaping post‐2025 public health governance. AMR, climate‐sensitive health threats, cross‐border surveillance, digital health governance, workforce migration and emergency preparedness increasingly require coordinated diplomatic action [23]. Regional institutions, including African health platforms, may play a growing role in negotiating equitable access to resources, strengthening preparedness and supporting collective resilience.

Although this framework remains conceptual, its practical value lies in offering policy directions that can guide implementation and future empirical validation. Comparative case studies, implementation research and evaluation of regional diplomatic initiatives are needed to determine which mechanisms most effectively advance equitable and sustainable health outcomes.

## 5 Conclusion

This article presents a conceptual framework, and therefore, does not empirically evaluate implementation outcomes. Future studies should validate these propositions through comparative case studies, implementation research and evaluation of regional HD initiatives.

HD in limited settings, particularly across Africa, is a crucial but complex endeavour that requires a deliberate shift from traditional aid‐based models to one of true partnership and equity. This article has utilized decolonial theory to expose how the persistent influence of hidden power sustains dependency and risks derailing well‐intentioned efforts. The urgency is underscored by the continent's interconnected health crises and the need for unified regional responses in surveillance, regulatory harmonization and resource pooling. Ultimately, achieving a realistic and equitable agenda demands more than just technical negotiation; it requires a commitment to strategic, values‐driven implementation. The path forward hinges on local ownership, HSS and a rigorous application of a rights‐based approach to actively mitigate power imbalances. By consciously adopting this framework, HD can transition from a tool that potentially reinforces existing inequalities to a powerful mechanism for building resilient, sustainable and truly equitable national and regional health security. This ensures health priorities reflect the needs of the community and the dignity of the individual, realizing health as a fundamental human right.

## Author Contributions

Majani Edward, Ritha Anderson Wililo and Godfrey Sama authored the original draft. Ntuli A. Kapologwe conceived the study design and revised the manuscript.

## Funding

The authors have nothing to report.

## Ethics Statement

The authors have nothing to report.

## Consent

The authors have nothing to report.

## Conflicts of Interest

The authors declare no conflicts of interest.

## Transparency Statement

The lead author, Majani Edward, affirms that this manuscript is an honest, accurate and transparent account of the study being reported; that no important aspects of the study have been omitted.

## Data Availability

The authors have nothing to report.
